# Clinical efficacy of unilateral laminotomy for bilateral decompression in the treatment of adjacent segment disease after lumbar fusion

**DOI:** 10.3389/fsurg.2024.1449838

**Published:** 2024-09-19

**Authors:** Yun Xu, Yang Liu, Ding Ding, Bin Ru, Quan Wan, Zhongwei Ji, Wenlong Liu, Ran Guo, Jiaqi Hu, Nannan Zhang, Langhai Xu, Shun Li, Wenjun Cai

**Affiliations:** ^1^ Department of Pain Management, Center for Rehabilitation Medicine, Zhejiang Provincial People’s Hospital, Affiliated People’s Hospital, Hangzhou Medical College, Hangzhou, Zhejiang, China; ^2^ Orthopedics and Traumatology Department, The Second Affiliated Hospital of Anhui University of Chinese Medicine, Hefei, Anhui, China

**Keywords:** unilateral laminotomy for bilateral decompression (ULBD), lumbar stenosis, adjacent segment disease, laminotomy, decompression

## Abstract

**Objective:**

To assess the clinical impact of unilateral laminotomy for bilateral decompression (ULBD) in managing patients with adjacent vertebrae following lumbar fusion.

**Methods:**

A retrospective analysis was conducted on 21 patients, with a mean age of 67.4 years, who underwent ULBD for adjacent vertebra disease at our department from January 2021 to November 2023. We reviewed demographic data, surgical techniques, imaging studies, and patient-reported outcomes. The study compared Visual Analog Scale (VAS) scores, Numeric Rating Scale (NRS) scores, Japanese Orthopaedic Association (JOA) scores, Short Form-36 (SF-36) scores, and imaging outcomes before surgery, immediately post-surgery, and at 1 month, 6 months, and 12 months post-surgery.

**Results:**

Evaluation of 21 patients with adjacent segment disease (ASD) (13 males, 8 females; mean age 67.42 years) was performed with follow-ups at various intervals post-surgery. Postoperative VAS, NRS, JOA, and SF-36 scores showed significant improvements compared to preoperative scores. Immediately after surgery, there were significant improvements in NRS score (2.76 ± 0.70 vs. 3.71 ± 0.85, *P* < 0.05) and JOA score (15.38 ± 1.02 vs. 9.29 ± 1.01, *P* < 0.05) compared to preoperative scores. Similarly, at 12 months post-surgery, significant improvements were observed in NRS score (1.52 ± 0.51 vs. 3.71 ± 0.85, *P* < 0.05) and JOA score (25.0 ± 1.10 vs. 9.29 ± 1.01, *P* < 0.05) compared to preoperative scores. The clinical satisfaction rate was 95.0% among all patients, with postoperative imaging examinations revealing a significant decompression effect. No complications were reported among the surgical patients.

**Conclusions:**

This study suggests that endoscopic ULBD can be a safe and effective technique for managing symptomatic ASD, providing satisfactory clinical outcomes for patients with ASD. Endoscopic ULBD may serve as an alternative treatment option for ASD with lumbar stenosis.

## 1 Introduction

Adjacent segment disease (ASD) refers to the emergence of new clinical symptoms associated with the cranial or caudal motion segment following lumbar spinal fusion. With the increasing number of patients undergoing spinal fusion surgery, the annual incidence of ASD exceeds 6% (1). Pathological processes contributing to ASD, which are instigated or accelerated by dynamic alterations in spinal mechanical stress, include disc herniation, osteophyte formation, facet joint hyperplasia, lateral recess stenosis, canal stenosis, vertebral instability, and spondylolisthesis. Zhong (2) et al. found that 98% of 18 patients with ASD exhibited cranial occurrence. Clinically, ASD can be categorized into three types (3): asymptomatic radiographic ASD, symptomatic ASD without radiographic changes, symptomatic ASD, and symptomatic ASD requiring surgical intervention.

ASD is often characterized by spinal stenosis, lateral recess stenosis, spinal instability, and other imaging abnormalities. The traditional surgical approach is typically posterior lumbar interbody fusion (PLIF), which involves extending the fusion segment after open decompression to provide adequate decompression and stability (4). However, scar tissue and altered anatomical structures at the previous surgical site increase the risks associated with PLIF. While some studies have reported favorable short-term outcomes with PLIF for treating ASD, they also noted a high long-term recurrence rate (5), underscoring the importance of addressing ASD recurrence. In recent years, spinal endoscopy techniques have advanced rapidly, offering benefits such as minimal trauma and rapid recovery. Murata et al. (6) suggested that endoscopic spinal decompression is an effective treatment for lumbar spinal stenosis caused by ASD, as it minimizes damage to bone and soft tissues while decompressing nerves, thereby reducing the incidence of iatrogenic spinal instability. In contrast, posterior endoscopic lumbar unilateral laminotomy for bilateral decompression (ULBD) can fully decompress the spinal canal and bilateral structures while preserving the normal stable structure of the lumbar spine (7).

In 1997, Spetzger et al. introduced the concept of unilateral laminectomy and bilateral decompression (ULBD) to minimize postoperative instability resulting from extensive decompression surgery (8). In 2002, Cuiot et al. advanced ULBD technology by reporting the use of endoscopic decompression through a unilateral surgical approach for treating degenerative lumbar spinal stenosis, based on Young's technique (9). ULBD is a minimally invasive technique that safely expands spinal canal volume, fully decompresses bilateral nerve roots and the dural sac, and minimizes damage to the integrity of the posterior spinal structure. It represents an ideal minimally invasive treatment for spinal stenosis with bilateral recess stenosis. Ba et al. (10) reported therapeutic outcomes with endoscopic decompression comparable to open extended posterior lumbar interbody fusion (PLIF) for treating adjacent vertebral diseases after lumbar fusion, with the endoscopic group benefiting from smaller surgical incisions and reduced blood loss. Wang et al. (11) observed that endoscopic surgery for adjacent vertebral diseases following spinal fusion could reduce the incidence of postoperative complications.

With the increasing incidence of adjacent vertebrae complications following lumbar fusion, a standardized approach to treatment is lacking. This study aims to investigate the clinical effectiveness of endoscopic ULBD in managing ASD post-lumbar fusion. By exploring the pathogenesis of adjacent vertebral diseases and the distinctive features of endoscopic ULBD technology, we seek to provide a comprehensive report on the outcomes of this surgical approach for clinical management.

## 2 Materials and methods

### 2.1 Patients

**Figure 1:**
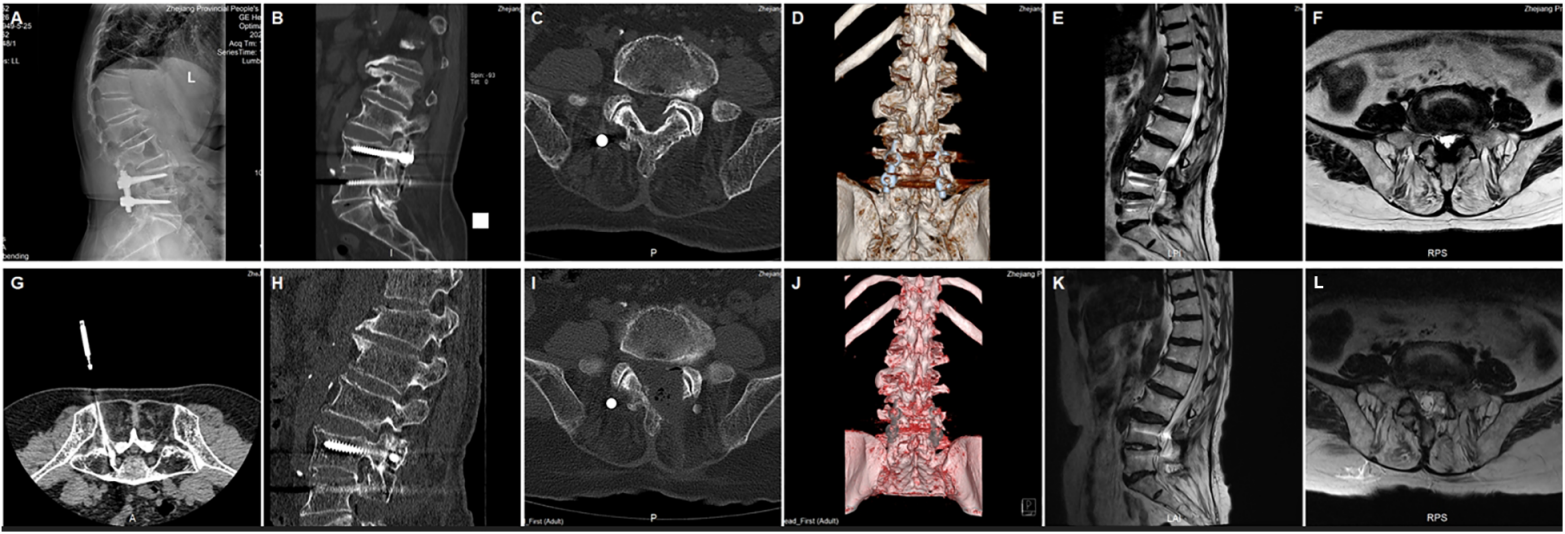
**(A)** Preoperative x-ray displaying spinal fusion **(B)** CT showing hyperplasia of small joints **(C)** CT showing stenosis of the lateral recess **(D)** preoperative CT three-dimensional reconstruction **(E)** preoperative T2-weighted MRI axial plane display of spinal canal stenosis **(F)** preoperative T2-weighted MRI sagittal plane display of spinal canal stenosis **(G)** Pre-surgery CT-guided selective nerve root block tests to pinpoint the responsible segment **(H)** CT showing removal of the small joint **(I)** CT showing removal of the left inferior lamina **(J)** CT three-dimensional reconstruction displaying inner edge small joint and partial removal of the vertebral plate **(K)** postoperative T2-weighted MRI sagittal plane **(L)** postoperative T2-weighted MRI axial plane.

This retrospective study focused on lumbar ASD patients treated with total endoscopic ULBD between January 2021 and November 2023. The research adhered to the principles of the Helsinki Declaration and underwent institutional review by our institution, approved by the Inspection Committee (IRB No. 1). All patients provided written informed consent. Inclusion criteria comprised symptomatic ASD patients post-lumbar fusion who had not responded to conservative treatments such as bed rest and physical therapy for at least 3 months. Exclusion criteria included lumbar spondylolisthesis, lumbar tumors, and adjacent segment infections. Twenty-one ASD patients following lumbar fusion met the inclusion criteria and were enrolled in the study (Figures 1A–F). All surgeries were performed by the same treatment team. Preoperatively, CT-guided selective nerve root block tests were conducted to identify the responsible segment (Figure 1G).

### 2.2 Surgical techniques

#### 2.2.1 Posture and anesthesia

All procedures were performed by the same team of physicians under dSA-guided epidural anesthesia combined with sedation using dexmedetomidine. The patient was positioned prone on the operating table, with an abdominal pad placed to elevate the lamina space and extend the ligamentum flavum, facilitating the procedure. The skin in the surgical area was thoroughly disinfected, and a waterproof membrane was applied. A smooth drainage system was established to allow the outflow of physiological saline, with the saline pressure controlled at 30 mmHg.

#### 2.2.2 Location and incision

On AP fluoroscopy, a vertical line was marked for the target segment, passing through the central points of the upper and lower pedicles. An approximately 8 mm skin incision was made at the midpoint of this vertical line. A series of soft tissue dilators were then inserted along the puncture guide needle into the target area, gradually expanding the surgical opening. Subsequently, the dilators were replaced by the working channel. Initial decompression was performed on the ipsilateral side. The caudal aspect of the superior vertebral lamina and any hyperplastic bony tissue were removed using an endoscopic high-speed grinding drill and ring saw. A portion of the ipsilateral ligamentum flavum was then separated after exposure. Basket forceps and upturned nucleus pulposus forceps were employed to remove the medial portion of the ligamentum flavum from the inferior articular process, revealing the compressed nerve roots and narrow lateral recess. After achieving full decompression of the ipsilateral recess and traversing root, the angle was adjusted to sequentially remove the joint between the lamina and spinous process, the base of the spinous process, and the ventral aspect of the contralateral lamina. The working channel was then adjusted to the dorsal side of the dural sac, and contralateral decompression was performed under microscopic guidance. Preservation of the deep layers of the ligamentum flavum was prioritized to safeguard the dural sac and underlying nerves. Basket forceps and upturned nucleus pulposus forceps were used to remove the contralateral ligamentum flavum from the contralateral recess, exposing the contralateral nerve roots. Following removal of the contralateral articular process joint and any associated small joint osteophytes using high-speed abrasive drills and laminoseter forceps under endoscopic visualization, the ligamentum flavum of the lateral recess was extracted with upturned nucleus pulposus forceps to achieve complete decompression of the contralateral compressed nerve roots and expansion of the lateral recess. Both lateral recesses and nerve roots were meticulously examined for any residual stenosis (Figures 1H–L). After achieving adequate hemostasis, a fixed drainage tube was inserted, and the surgical incision was sutured.

### 2.3 Evaluation indicators

The collected clinical parameters included operation time, length of stay, fluoroscopic time, and MacNab score. NRS and VAS were used to assess waist and leg pain in the enrolled patients before surgery, immediately post-surgery, and at 1 month, 6 months, and 12 months post-surgery. The Japanese Orthopaedic Association (JOA) score was employed to evaluate clinical outcomes before surgery and at the 12-month follow-up, with modifications. The improvement rate was calculated as follows: improvement rate (%) = (postoperative JOA score—preoperative JOA score)/(29—preoperative JOA score). The Short Form-36 (SF-36) questionnaire, comprising 36 items, was used to evaluate patients’ quality of life. The SF-36 encompasses 8 dimensions: Physical Functioning, Physical Role, Physical Pain, General Health, Vitality, Social Functioning, Emotional Role, and Mental Health. Higher scores across all eight dimensions indicate a better quality of life.

### 2.4 Statistical analysis

All statistical analyses were performed using SPSS 21.0. Data were expressed as mean ± standard deviation. The paired Student's *t*-test was used to compare preoperative and postoperative continuous variables, including VAS, NRS, JOA, and SF-36. All *P* values were bilateral, with *P* < 0.05 considered statistically significant.

## 3 Results

### 3.1 Demographic statistics

A total of 21 patients with ASD were enrolled in this study, comprising 13 males and 8 females, with a mean age of 67.43 ± 8.12 years. The mean duration of disease was 28.34 ± 10.12 months. Lesions were distributed as follows: L3-L4 (6 cases), L4-L5 (2 cases), and L5-S1 (13 cases) (Table 1). Follow-up evaluations were conducted at 1 month, 6 months, and 12 months postoperatively.

**Table 1:** Demographic characteristics.

Variable	Values
Sex	
Male	13 (61.9%)
Female	8 (38.1%)
Age, years, Mean ± SD (range)	67.43 ± 8.12
BMI, kg/m^2^, Mean ± SD (range)	20.76 ± 1.38
Course of disease, months, mean ± SD (range)	28.76 ± 1.51
Diagnosis	
Central canal stenosis	
Lateral recess stenosis	
Segment distribution	
L3–L4	6 (28.6%)
L4–L5	2 (9.5%)
L5-S1	13 (61.9%)
Comorbidities	
Hypertension	9 (42.9%)
Diabetes	8 (38.1%)
Cardiac disease	5 (23.8%)

### 3.2 Clinical parameters and complications

The mean operation time was 70.38 ± 1.00 min, and the mean fluoroscopy time was 26.86 ± 1.46 min (Table 2). Postoperative imaging revealed a 100% retention rate of facet joints, with a significant increase in the spinal canal area from 69.95 ± 7.86 mm² to 160.38 ± 10.41 mm² (Table 3). The mean length of hospital stay was 5.52 ± 0.73 days. According to the MacNab classification of patient satisfaction, 7 patients had excellent outcomes, 11 had good outcomes, 2 had fair outcomes, and 1 had a poor outcome, resulting in an overall satisfaction rate of 95.0%. No cases of epidural tear, epidural hematoma, or postoperative infection were reported (Table 4).

**Table 2:** Clincal outcomes.

Variables	Values
Operative time, minutes, mean ± SD	70.38 ± 1.00
Fluoroscopy, seconds, mean ± SD	26.86 ± 1.46
Hospital stay, days, mean ± SD	5.52 ± 0.73
MacNab calssification of satisfaction	
Excellent	7
Good	11
Fair	2
Poor	1

**Table 3:** Radiological outcomes.

Enlarged area of spinal canal (mm^2^)	
Preoperative	69.95 ± 7.86
Postoperative	160.38 ± 10.41
Joint retention rate of articular processes (%)	100

**Table 4:** Complications.

Dural tear	0
Postoperative dysesthesia	0
Recurrence	0

### 3.3 Assessment of patient quality of life

Postoperative VAS, NRS, and JOA scores showed significant improvements compared to preoperative scores. Immediately after surgery, there were significant improvements in NRS score (2.76 ± 0.70 vs. 3.71 ± 0.85, *P* < 0.01) and JOA score (15.38 ± 1.02 vs. 9.29 ± 1.01, *P* < 0.01) compared to preoperative scores. Similarly, at 12 months post-surgery, significant improvements were observed in NRS score (1.52 ± 0.51 vs. 3.71 ± 0.85, *P* < 0.01) and JOA score (25.0 ± 1.10 vs. 9.29 ± 1.01, *P* < 0.01) compared to preoperative scores (Table 5). The SF-36 questionnaire was used to assess patients’ quality of life, encompassing both physical components (physical function, role physical, and bodily pain) and mental components (general health, vitality, social functioning, role emotional, and mental health) before surgery and at the latest follow-up (Table 6).

**Table 5:** Patient-Reported outcomes.

Variable	Prep.	1-Day Postop	3-Month Postop	6-Month Postop	12-Month Postop
VAS for back pain	4.29 ± 0.96	3.48 ± 0.60	2.10 ± 0.70	1.76 ± 0.54	1.43 ± 0.51
VAS for leg pain	8.38 ± 1.16	5.10 ± 0.83	3.10 ± 0.83	2.52 ± 0.51	1.57 ± 0.51
NRS	3.71 ± 0.85	2.76 ± 0.70	1.95 ± 0.67	1.76 ± 0.54	1.52 ± 0.51
JOA	9.29 ± 1.01	15.38 ± 1.02	17.43 ± 1.02	19.57 ± 1.34	25.0 ± 1.10

**Table 6:** SF-36.

PF	44.29 ± 1.19	59.67 ± 1.83	74.05 ± 0.97	80.10 ± 1.26
RP	22.24 ± 1.22	44.19 ± 1.70	59.62 ± 1.02	68.90 ± 1.22
BP	30.24 ± 1.41	56.10 ± 1.64	59.62 ± 1.02	69.90 ± 1.34
GH	55.86 ± 1.65	59.24 ± 1.22	62.14 ± 1.15	63.57 ± 1.20
VT	44.14 ± 1.35	67.14 ± 1.11	76.57 ± 1.21	79.65 ± 1.10
SF	50.24 ± 1.48	69.57 ± 1.33	73.05 ± 1.24	78.33 ± 1.35
RE	51.05 ± 1.28	76.62 ± 1.32	79.29 ± 1.38	81.33 ± 1.07
MH	68.19 ± 1.29	69.52 ± 1.25	71.14 ± 1.24	73.29 ± 1.27

## 4 Discussion

Currently, the treatment options for lumbar ASD vary, ranging from endoscopic to open surgery. Clinicians aim to achieve adequate decompression and rapid recovery while preserving lumbar spine stability when addressing peripheral vertebral diseases. However, there are limited clinical reports comparing the efficacy of ULBD in managing adjacent vertebral diseases. This study retrospectively analyzed the clinical data of 21 patients with adjacent vertebral disease following lumbar fusion in our department to explore the clinical efficacy of ULBD for treating adjacent vertebral disease post-lumbar fusion.

Peripheral vertebropathy often emerges as a mid- to long-term complication in patients after spinal fusion, particularly following multi-segment spinal fusion (12). It typically manifests at the cranial end of adjacent segments to the fused vertebrae. Post-lumbar fusion, the strength of the fusion segment increases, redistributing spine motion during flexion or extension activities, which necessitates increased involvement of the cranial end in compensatory movements (13). The goal of surgical management is to restore normal physiological function by relieving compression on the spinal cord, nerves, and blood vessels. While open internal fixation fusion offers a definitive therapeutic outcome, the associated surgical trauma, extensive soft tissue dissection, and significant blood loss often lead to postoperative lumbar pain and muscle atrophy (14). Alterations in spinal biomechanics, damage to the ligament complex, and intervertebral disc degeneration after surgery contribute to the increased susceptibility to degeneration observed in adjacent segments on imaging examinations.

Complications such as spinal canal stenosis, vertebral instability, lateral recess stenosis, and facet joint dysplasia are common. However, the intermediate and long-term efficacy of current treatments for these conditions remains suboptimal. Patients with such spinal degeneration often experience significant clinical symptoms due to nerve compression and spinal cord injury, which adversely affects their quality of life and reduces satisfaction following spinal surgery. Therefore, analyzing methods to reduce the incidence of adjacent vertebral disease after lumbar fusion and enhance postoperative efficacy has become increasingly important, drawing greater attention from clinicians. Common risk factors for ASD include the length of the fusion segment, extent of laminectomy, soft tissue injury, and internal fixation.

For patients with ASD, conservative treatment is typically recommended initially, with surgical intervention considered if conservative measures fail to alleviate symptoms for more than 3 months. A retrospective analysis of ASD incidence among 751 patients undergoing initial lumbar discectomy reported a reoperation rate as high as 10% (15). Revision open surgery is often the standard choice for conservative treatment failure (16), though it carries a risk of iatrogenic spinal instability. Fusion of the unstable vertebral body during surgery sacrifices spinal motion and accelerates degeneration of adjacent intervertebral discs. Consequently, there is a pressing need for spinal surgeons to identify surgical approaches that minimize damage to surrounding spinal tissues while ensuring overall spinal stability and surgical efficacy. The concept of reducing structural spinal damage has gained prominence, leading to the introduction of minimally invasive spinal surgery. Endoscopic spine surgery, which features small incisions and short operation times, minimizes damage to the paraspinal muscle group and disrupts stable posterior structures minimally, thereby maximizing lumbar spine stability. Surgeons can perform nerve decompression and spinal canal expansion under endoscopic guidance, often using local anesthesia, allowing continuous communication with patients during the procedure and effectively reducing the risk of nerve injury. With high surgical precision, endoscopic spine surgery reduces postoperative complication rates, enabling patients to resume activities early and return swiftly to normal life, including work.

Percutaneous transforaminal endoscopic discectomy (PTED) has become a widely utilized treatment modality for lumbar degenerative diseases. In treating patients with ASD, Gu et al. (17) conducted a retrospective evaluation of 25 ASD patients aged over 65 years who underwent PTED. Among these patients, 84.0% experienced good or satisfactory clinical outcomes, 12.0% reported fair outcomes, and 4.0% indicated poor outcomes. Postoperative assessments revealed improvements in VAS, ODI, and JOA scores compared to preoperative scores. These findings suggest that PTED effectively alleviates pain associated with ASD in elderly patients, with favorable surgical safety and postoperative recovery. Similarly, Kapetanakis et al. also supported the efficacy of PTED in enhancing postoperative quality of life for ASD patients, considering it an effective surgical option (18). However, Li Jie et al. (19), while confirming the efficacy of PTED in ASD treatment, raised concerns about biomechanical alterations due to excessive excision of the superior articular process during surgery, highlighting that loss of this process may induce spinal instability.

As early as 1988, Young pioneered the hemilaminectomy technique, performing contralateral spinal canal, lateral recess, and foraminal decompression using a unilateral laminar space approach under a microscope. This technique preserved the contralateral articular process structure while allowing exploration of the contralateral pedicle for maximal decompression. By dissecting only one side of the paravertebral soft tissue and preserving the contralateral ligament complex and deep muscles, the technique minimized contralateral soft tissue injury. Endoscopic ULBD merges the advantages of open and endoscopic spine surgery. It allows for bilateral decompression of the dural sac and nerve roots through a unilateral approach while preserving the posterior spinal structure. Typically, decompression on the operative side involves removing the lower two-thirds of the upper vertebral lamina and the upper one-third of the lower vertebral lamina, while retaining the isthmic part of the lateral lamina and a single cortical layer on the contralateral side to maintain spinal motion and stability, thus avoiding iatrogenic instability. Microendoscopic spinal decompression is also effective for various refractory lumbar spinal stenosis cases requiring decompression (20). However, using air as a medium with microscopes and microscopic endoscopy may impair the clarity of the field, particularly in contralateral decompression surgeries (21). Additionally, microscopic ULBD might require relatively extensive muscle and laminectomy, while endoscopic ULBD achieves decompression with reduced intraoperative bone and soft tissue damage. Nevertheless, ULBD has limitations. Patients with spinal instability might experience recurrent postoperative pain due to disc height recovery. In cases of laminar space stenosis and ossification of the posterior longitudinal ligament, decompression might compromise facet joint stability, increasing the risk of nerve root injury (22).

In our study, significant improvements were observed in VAS, NRS, JOA scores, and SF-36 index values postoperatively, with sustained enhancement over time. Postoperative imaging reviews confirmed complete decompression.

All surgeries in this study were performed under epidural anesthesia, which effectively alleviated surgery-related anxiety in ASD patients and facilitated their psychological acceptance of treatment. Furthermore, maintaining communication with awake patients allowed surgeons to effectively reduce the risk of nerve damage. Despite this, ASD patients, who are often older and have comorbidities such as diabetes and hypertension, may exhibit poor surgical tolerance and a higher incidence of complications. None of the patients in this study experienced significant exacerbation of preexisting medical conditions after surgery.

Complications of endoscopic spine surgery that concern clinicians generally include dural and nerve root injuries, with an incidence ranging from 3% to 14%. Lin et al. (23) reported nine surgical complications among 127 patients with lumbar spinal stenosis treated with unilateral laminotomy under endoscopy, including five cases of dural tears and four cases of epidural hematoma. Intraoperative adhesion between the dura and ligamentum flavum can lead to dural damage during ligamentum flavum stripping, resulting in cerebrospinal fluid leakage and epidural hematoma, which adversely affects patient prognosis (24). To minimize these occurrences, meticulous surgical technique is essential. Additionally, preoperative use of anticoagulant drugs should be reviewed, and preoperative coagulation indicators assessed. Bone margins and the epidural space are carefully inspected under low irrigation pressure before suturing, and closed drainage is routinely applied for 48 h postoperatively.

## 5 Limitations

This study has several limitations. The patient cohort was small, and the follow-up duration was relatively short. Additionally, there was no control group included. Furthermore, the study was restricted to patients with single-segment stenosis, limiting the generalizability of the findings to cases of multisegment stenosis.

## 6 Conclusion

Endoscopic ULBD shows potential benefits, including improvements in postoperative VAS, NRS, and JOA scores, as well as a reduction in complication rates for patients with adjacent vertebrae. It may represent an effective and suitable treatment option for peripheral vertebral diseases.
