# Corrigendum: Clinical efficacy of unilateral laminotomy for bilateral decompression in the treatment of adjacent segment disease after lumbar fusion

**DOI:** 10.3389/fsurg.2024.1499570

**Published:** 2024-10-29

**Authors:** Yun Xu, Yang Liu, Ding Ding, Bin Ru, Quan Wan, Zhongwei Ji, Wenlong Liu, Ran Guo, Jiaqi Hu, Nannan Zhang, Langhai Xu, Shun Li, Wenjun Cai

**Affiliations:** ^1^ Department of Pain Management, Center for Rehabilitation Medicine, Zhejiang Provincial People’s Hospital, Affiliated People’s Hospital, Hangzhou Medical College, Hangzhou, Zhejiang, China; ^2^ Orthopedics and Traumatology Department, The Second Affiliated Hospital of Anhui University of Chinese Medicine, Hefei, Anhui, China

**Keywords:** unilateral laminotomy for bilateral decompression (ULBD), lumbar stenosis, adjacent segment disease, laminotomy, decompression

A corrigendum on Clinical efficacy of unilateral laminotomy for bilateral decompression in the treatment of adjacent segment disease after lumbar fusion By Xu Y, Liu Y, Ding D, Ru B, Wan Q, Ji Z, Liu W, Guo R, Hu J, Zhang N, Xu L, Li S, Cai W (2024). Front. Surg. 11:1449838. doi: 10.3389/fsurg.2024.1449838

**Incorrect Article Type**

In the published article, there was an error in the article type. Instead of “Review”, it should be “Original Research”.

The authors apologize for this error and state that this does not change the scientific conclusions of the article in any way. The original article has been updated.

